# Correction: TET3 is a positive regulator of mitochondrial respiration in Neuro2A cells

**DOI:** 10.1371/journal.pone.0300015

**Published:** 2024-02-29

**Authors:** The PLOS ONE Staff

## Notice of Republication

This article was republished on February 15, 2024, to correct errors in the figures. Figure 2 and Figure 3 were inadvertently swapped, and Figure 4 was incorrectly published. The article’s Data Availability statement has also been updated. The publisher apologizes for these errors. Please download this article again to view the correct version. The originally published, uncorrected article and the republished, corrected articles are provided here for reference.

## Supporting information

S1 File Originally published, uncorrected article. (PDF)

S2 File Republished, corrected article. (PDF)
