# Supplementary material for: TET3 is a positive regulator of mitochondrial respiration in Neuro2A cells
Source: PLoS One. 2024 Jan 16;19(1):e0294187. doi: 10.1371/journal.pone.0294187 (PMC10790995; doi:10.1371/journal.pone.0294187)
Supplement: S1 File — (PDF) [file pone.0294187.s001.pdf]

Supporting Information

S1 Table. Primers used in QPCR Analyses

Gene	Forward primer 5’-3’	Reverse primer 5’-3’
Agtpb1	TCCTGCCACCCGGATTATCT	GAGTCCCACAACCCGAGAAC
Atp5e	TGAGGCTACTCTGAAGCGAC	TCTTCAGGGCATCCCTCACT
Atp5mg	GGCCACATTTTGGCACTACG	GTGGCCACCAAACCATTCAG
Canx	TTCCAGACCCTGATGCAGA	TCCCATTCTCCGTCCATATC
Endog	CCAACCACCGCTGGAGTCA	GACTTCCCATCAGCCTCGG
Higd2a	CAAGACCCGTGAGAACCCAA	TACGACCGTGAAACCCTGTG
Isca2	GTGGATGCTCCGGATTCCAA	TTGCCCCACCCTGTTCAAAT
mtCo1	TCGGAGCCCCAGATATAGCA	TTTCCGGCTAGAGGTGGGTA
mtCo2	AACCGAGTCGTTCTGCCAAT	CTAGGGAGGGGACTGCTCAT
mtNd1	TCCGAGCATCTTATCCACGC	GTATGGTGGTACTCCCGCTG
mtNd3	GTTGCATTCTGACTCCCCCA	GGTAGACGTGCAGAGCTTGT
Ndufb8	CGTGTTCCCTTCCTACCAGC	GGCAAAAAGCCCATCAAGCC
Pdk1	GCCAGGTGGACTTCTATGCG	AGGCAACTCTTGTCGCAGAA
Pdpr	CTGGGCTGCCCTTCACTTC	TCACCACCTGGAAGATACGTT
Tet1	GAGCCTGTTCCTCGATGTGG	CAAACCCACCTGAGGCTGTT
Tet2	TGTTGTTGTCAGGGTGAGAATC	TCTTGCTTCTGGCAAACTTACA
Tet3	CCGGATTGAGAAGGTCATCTAC	AAGATAACAATCACGGCGTTCT
Tmem65	GGGTTTGGCCTCCTACGG	TGCCCAGACAGGTTTTCTGC
Uqcr10	CTGGGCCATCCTGACCTTG	ACTCCAGGCAAACAGCTGAC

S1 Fig. An example of a gating strategy used to identify TMRE+ N2a cells. (A) Fixable viability stain 780 and TMRE dye were used to identify live cells and mitochondrial potentiation, respectively, in N2a cells. (B) N2a cells without TMRE treatment were used to confirm TMRE staining is specific. (C) N2a cells were treated with TMRE dye together with FCCP (uncoupling agent) as a negative control to gate TMRE positive cells. There was approximately 20% less TMRE positive cells in the TMRE+FCCP treated cells compared to the cells treated only with TMRE. (D) Representative histogram of siCtr and siTet3 demonstrating a decrease in TMRE abundance as indicated by a reduction in Mean Fluorescence Intensity (MFI).

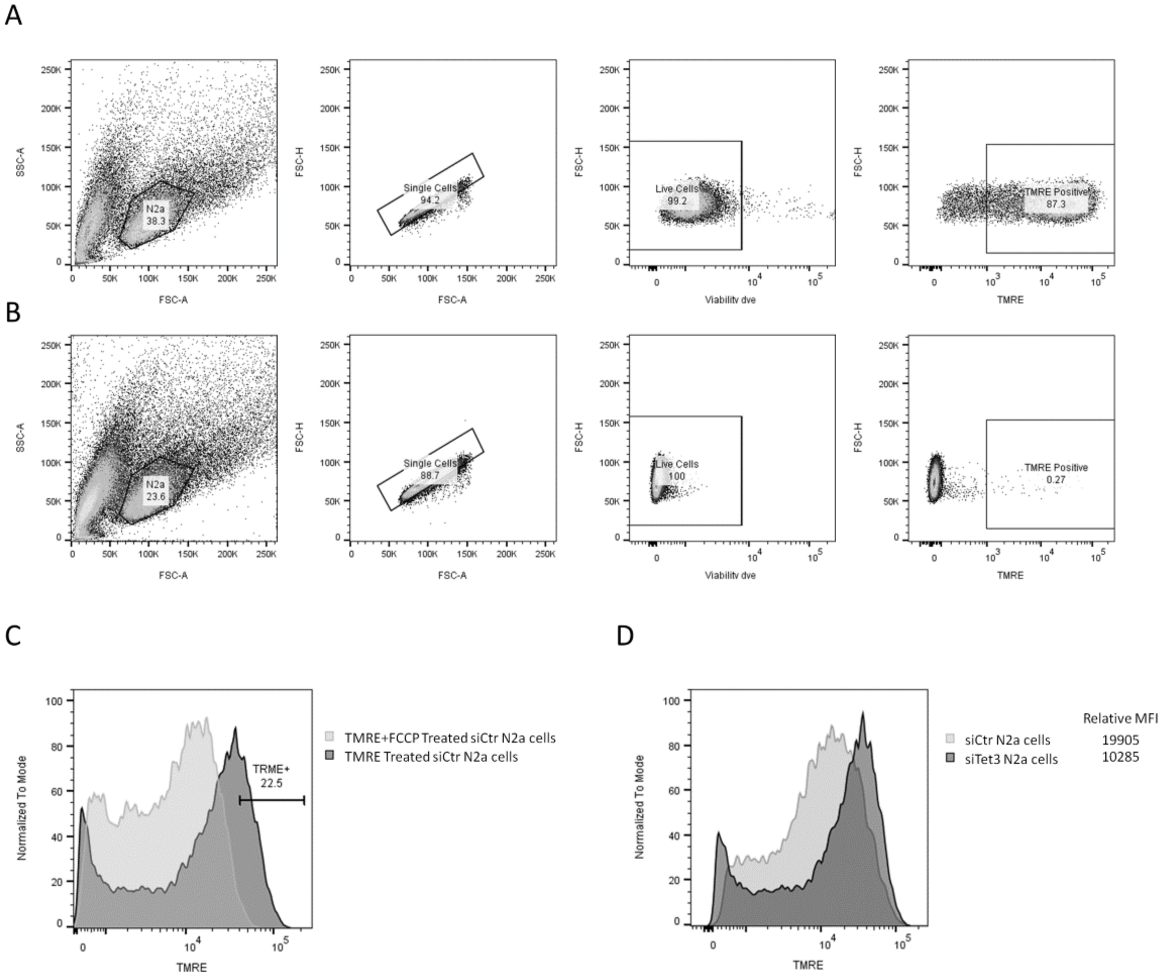

S2 Fig. TET3 is responsible for significant 5-hmC production in N2a cells. Global levels of 5-hmC in two different representative experiments of N2a cells in which TET3 was silenced for 72 hours and in negative control siRNA-transfected N2a cells were determined by immune dot-blot as in previous studies (Burr et al., *Nucleic Acids Research* (2018). 46 (3), 1210–1226.)

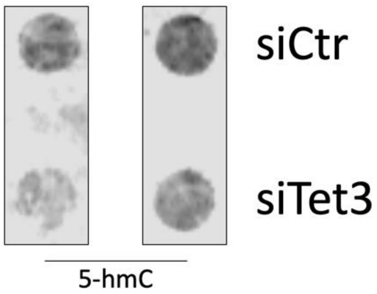

S3 Fig. Glycolytic stages of siCtr and siTet3 treated N2a cells. (A) Tet3-silenced cells exhibited no changes in glycolysis and glycolytic capacity (n=13 including 3 biological replicates). (B) Energy map demonstrates a change in oxygen consumption rate but no changes in extra cellular acidification rate between the two cell populations**.**

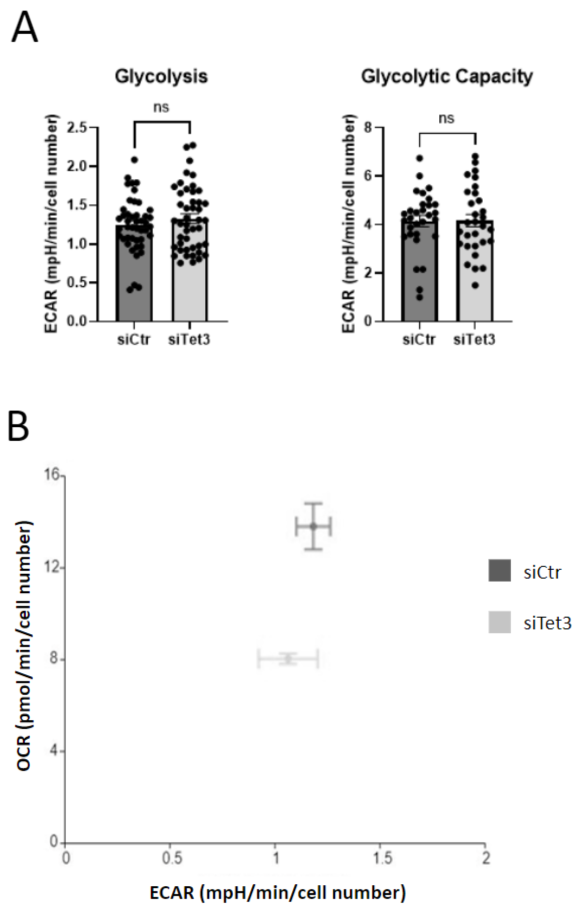

S4 Fig. No 5hmC marks were detected on the Epi P1 region. The sequence of a region of Epi P1 after amplification from Tet3-silenced or control N2a cells (as depicted in Figure 4C). 5hmC residues within N2A genomic DNAs were protected from deamination by APOBEC, by conversion to glucosylated-hydroxymethylcytosine by treatment with T4 Phage β-glucosyltransferase. There was no difference observed between siCtr and siTet3, thus no 5hmC mark was detected.

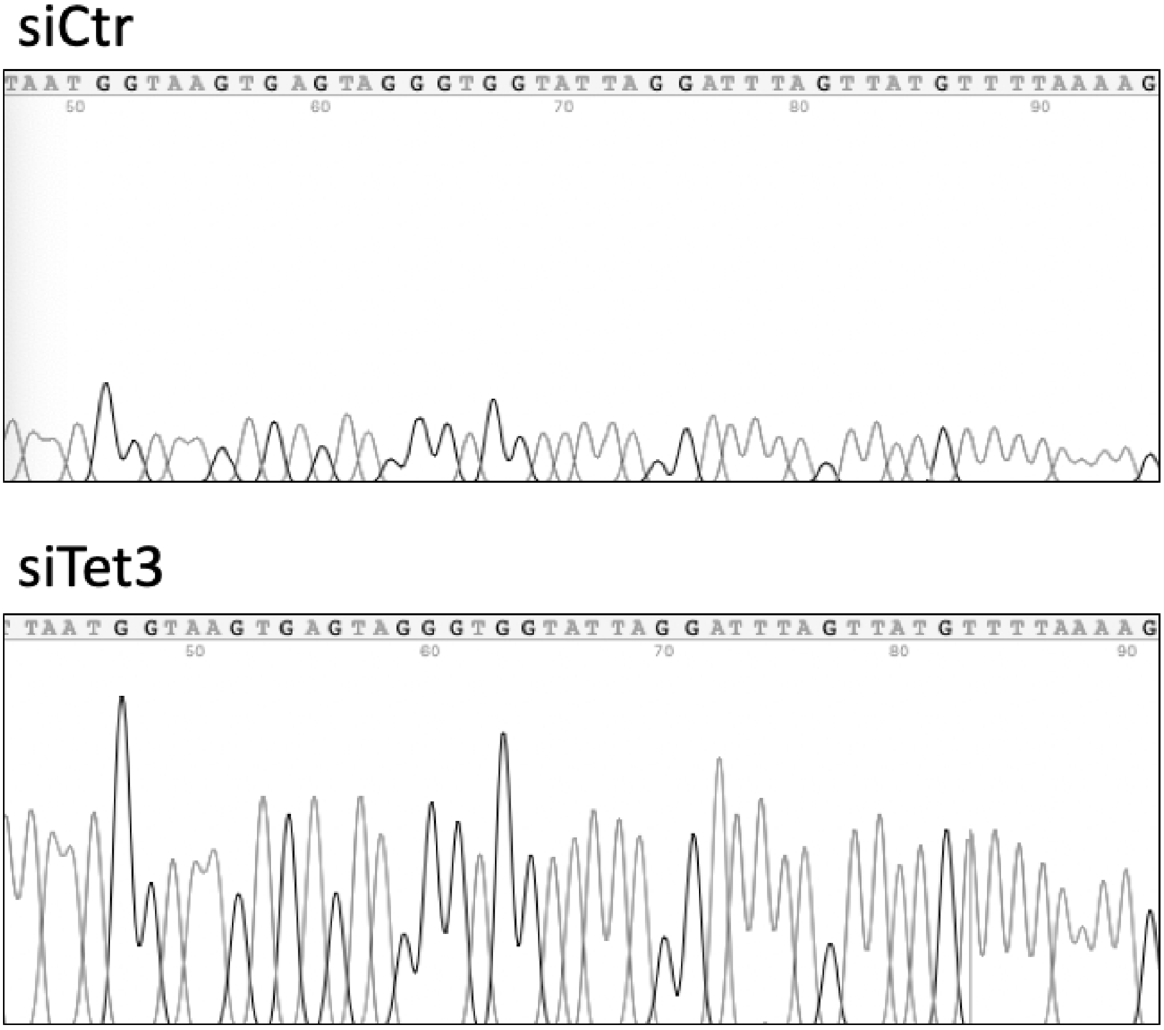

S2 Table. **List of genes upregulated in TET3-silenced, compared to control N2A cells.** Genes with a statistical significance of P1≤0.0001 are listed. FDR: false discovery rate.

Symbol	log2FoldChange (N2aTET3/N2aControl)	FDR	Pvalue
Mgp	9.812177	1.63E-25	6.72E-27
Matn1	8.960002	1.97E-48	4.22E-50
3110079O15Rik	8.921841	3.68E-10	3.91E-11
Rhox2b	8.764872	2.46E-15	1.73E-16
Gm10487	8.276124	4.96E-13	4.15E-14
Hapln1	7.930737	8.33E-61	1.35E-62
Gm14430	7.918863	9.79E-54	1.87E-55
Igf2	7.758223	0	0
Col3a1	7.72792	3.7E-58	6.44E-60
Mt2	7.672425	2.43E-05	4.46E-06
Gm2012	7.622052	3.66E-08	4.73E-09
H2-Q8	7.442943	3.66E-08	4.73E-09
Gm5072	7.266787	1.35E-07	1.86E-08
Fibin	7.238405	4.78E-15	3.43E-16
Col1a2	7.238405	3.8E-187	1.6E-189
Scrg1	7.238405	4.62E-05	8.85E-06
Hist2h2aa2	7.199672	0.000596	0.000137
Ppic	7.189825	5.12E-09	6.06E-10
Col5a2	7.159871	1.17E-46	2.57E-48
Col5a1	7.066089	8.46E-56	1.54E-57
Col6a2	6.960002	1.35E-43	3.14E-45
Epyc	6.894818	7.06E-10	7.76E-11
Ogn	6.807355	6.69E-26	2.7E-27
Comp	6.796271	1.68E-87	1.75E-89
Thbs1	6.774787	2E-119	1.5E-121
Col2a1	6.701329	0	0
Fmod	6.686501	4.33E-81	4.95E-83
Abi3bp	6.523562	5.65E-20	3.01E-21
Ctgf	6.510962	3.92E-50	8.07E-52
Penk	6.409391	7.04E-22	3.38E-23
Clec3a	6.392317	6.99E-12	6.42E-13
Col11a1	6.294621	2.3E-276	5.1E-279
Ccnd2	6.247928	6.98E-20	3.75E-21
Smoc2	6.247928	1.92E-10	1.97E-11
6230416C02Rik	6.209453	8.79E-05	1.75E-05
Col9a1	6.192019	2.6E-111	2.1E-113
Col6a3	6.149747	7.97E-33	2.46E-34

Sdc2	6.066089	9.92E-11	9.94E-12
Nid2	6.066089	6.48E-16	4.4E-17
Plagl1	6.055282	4.42E-31	1.45E-32
Mmp14	6.044394	3.61E-12	3.24E-13
H19	6.028354	5.1E-246	1.3E-248
Zcchc5	5.882643	9.86E-09	1.2E-09
Lox	5.857981	2.57E-13	2.09E-14
Oas1g	5.83289	2.43E-05	4.46E-06
Ppp1r1b	5.83289	1.28E-05	2.25E-06
Ccdc80	5.83289	9.61E-19	5.5E-20
Acan	5.815063	2.1E-184	8.7E-187
Ptx3	5.643856	8.79E-05	1.75E-05
Fgfr2	5.61471	6.98E-12	6.42E-13
Tspan6	5.584963	0.000317	0.000069
Frzb	5.554589	9.59E-07	1.45E-07
Ucma	5.46307	4.65E-15	3.33E-16
Hist1h2br	5.364572	4.65E-15	3.33E-16
Hist1h2bq	5.364572	4.65E-15	3.33E-16
Tbx15	5.357552	4.98E-07	7.32E-08
Aspn	5.357552	0.000088	1.75E-05
Spp1	5.330917	9.21E-10	1.02E-10
Synpo	5.321928	7.07E-10	7.76E-11
Emp2	5.321928	9.59E-07	1.45E-07
Cd109	5.285402	2.64E-11	2.53E-12
Myct1	5.285402	2.43E-05	4.46E-06
Mmp2	5.209453	9.21E-10	1.02E-10
Col1a1	5.153805	1.9E-222	6.4E-225
Tmem45a	5.129283	8.79E-05	1.75E-05
Postn	5.129283	1.28E-05	2.25E-06
Itih5l-ps	5.087463	2.6E-07	3.69E-08
Tnfrsf11b	5.087463	0.000088	1.75E-05
Enpp2	5.087463	1.28E-05	2.25E-06
Tnc	5.044394	4.87E-10	5.27E-11
Padi6	5.044394	0.000596	0.000137
Col8a2	5.044394	5.85E-06	9.81E-07
Pcolce2	4.954196	2.66E-07	3.78E-08
Xylt1	4.954196	0.000088	1.75E-05
Bgn	4.926654	2.04E-87	2.13E-89
Edil3	4.906891	7.05E-08	9.38E-09
Csgalnact1	4.906891	6.7E-06	1.13E-06
Srpx2	4.857981	0.000317	0.000069
Col10a1	4.857981	5.14E-15	3.7E-16
Loxl1	4.857981	8.79E-05	1.75E-05
Slc7a2	4.83289	2.39E-18	1.4E-19

Col12a1	4.790077	2.29E-40	5.72E-42
Lum	4.754888	4.95E-07	7.27E-08
Stk26	4.754888	0.000167	3.48E-05
Chrdl1	4.754888	1.28E-05	2.25E-06
Cd99	4.73954	2.66E-07	3.78E-08
Rftn2	4.70044	0.000088	1.75E-05
Timp3	4.698126	4.7E-117	3.7E-119
Acox2	4.672425	1.97E-05	3.57E-06
Tns3	4.643856	5.12E-09	6.06E-10
Prss23	4.584963	0.000595	0.000137
Grb10	4.523562	1.03E-21	4.99E-23
Kirrel	4.523562	4.95E-07	7.27E-08
Kcna6	4.523562	1.84E-06	2.88E-07
Nrk	4.523562	1.35E-07	1.86E-08
Thbd	4.392317	0.000596	0.000137
Yap1	4.392317	0.000167	3.48E-05
Myo1d	4.392317	0.00012	2.45E-05
Gm5860	4.343408	6.16E-09	7.38E-10
Tgfbr2	4.273018	6.42E-11	6.31E-12
Fbln5	4.247928	0.00012	2.45E-05
Thbs2	4.247928	1.28E-05	2.25E-06
Lect1	4.241367	2.16E-59	3.64E-61
Osmr	4.169925	0.000167	3.48E-05
Itpripl2	4.169925	3.52E-06	5.71E-07
H6pd	4.087463	0.000596	0.000137
Smo	4.087463	9.93E-08	1.34E-08
Plekhh2	4.087463	1.28E-05	2.25E-06
Atp11a	4.087463	3.53E-06	5.71E-07
Boc	3.938599	9.93E-08	1.34E-08
Fbn2	3.906891	7.05E-08	9.38E-09
Phldb2	3.906891	0.000317	0.000069
Col9a2	3.841302	3.45E-37	9.32E-39
Procr	3.793256	6E-151	3.4E-153
Prelp	3.777608	2.47E-12	2.17E-13
Lmo7	3.70044	0.000596	0.000137
Vldlr	3.70044	2.43E-05	4.46E-06
Adamtsl3	3.70044	0.000708	0.000165
Crispld1	3.70044	0.000397	8.78E-05
S100b	3.684498	2.04E-05	3.7E-06
Dab2	3.643856	0.00012	2.45E-05
Cgnl1	3.584963	0.000317	0.000069
Adgrg6	3.584963	0.000596	0.000137
Cp	3.584963	3.63E-05	6.83E-06
Col27a1	3.584963	2.98E-09	3.44E-10

Serpinh1	3.498707	2.14E-90	2.12E-92
Lcn2	3.435386	1.05E-06	1.59E-07
Sorbs2	3.426265	1.38E-05	2.45E-06
Mfi2	3.392317	1.01E-05	1.75E-06
Omd	3.345775	3.29E-07	4.74E-08
Svil	3.321928	0.000596	0.000137
Svep1	3.321928	6.71E-06	1.13E-06
Cdkn1c	3.095157	1.14E-10	1.15E-11
Trpv4	3	5.35E-05	1.03E-05
Col9a3	2.816161	1.83E-36	5.11E-38
Papss2	2.677023	4.4E-25	1.83E-26
4933432K03Rik	2.612654	0.000327	7.14E-05
Cspg4	2.608809	4.85E-12	4.38E-13
Cilp	2.603341	2.4E-08	3.02E-09
Lgals3	2.548893	7.88E-11	7.82E-12
Itm2a	2.524117	1.15E-24	4.88E-26
Alms1-ps2	2.494765	4.47E-07	6.53E-08
1700026L06Rik	2.487266	0.000894	0.000213
Pcdha4	2.459432	2.58E-06	4.11E-07
Igsf10	2.321928	3.05E-07	4.37E-08
Scara3	2.311944	7.69E-11	7.63E-12
Dcn	2.238405	3.8E-06	6.19E-07
Hist1h2bc	2.215869	4.32E-19	2.43E-20
Gm17026	2.164745	0.000146	3.02E-05
4930486L24Rik	2.153474	2.43E-06	3.87E-07
Gcnt4	2.152003	2.12E-16	1.39E-17
Cryba2	2.147754	0.000978	0.000235
Gm7367	2.11456	3.62E-14	2.78E-15
Snx33	2.109122	4.78E-26	1.92E-27
Hist2h2bb	2.105353	1.54E-05	2.75E-06
D330041H03Rik	2.077444	2.45E-08	3.09E-09
Elf3	2.075288	6.17E-05	0.000012
Akr1b7	2.046474	1.53E-08	1.89E-09
Amd2	2.017922	2.44E-05	4.49E-06
Ssxb3	2.012278	2.42E-05	4.43E-06
Loxl4	2	3.75E-09	4.39E-10
Gcnt2	1.9855	6.16E-08	8.13E-09
Smpd5	1.923379	2.34E-07	3.32E-08
Ptgds	1.912537	2.11E-14	1.59E-15
Aim1l	1.906891	3.47E-05	6.51E-06
Otog	1.903324	3.16E-17	1.97E-18
Nptx1	1.887525	0.000883	0.00021
Wnt5b	1.884523	0.000576	0.000132
Fhit	1.876128	0.000141	2.91E-05

Lrrc71	1.874469	0.000967	0.000232
Slc16a12	1.871584	1.94E-41	4.73E-43
Ropn1l	1.856767	8.69E-05	1.73E-05
Slco2b1	1.851477	6.94E-06	1.17E-06
5830444B04Rik	1.83289	2.38E-06	3.78E-07
1810010H24Rik	1.832106	2.1E-21	1.04E-22
Chrnb1	1.82103	1.26E-07	1.73E-08
Prdm8	1.819428	9.42E-18	5.73E-19
Nos1	1.785102	1.73E-28	6.32E-30
Apol9b	1.777608	1.29E-06	1.99E-07
Nqo1	1.765535	5.53E-12	5.02E-13
Vcam1	1.76356	6.81E-08	9.03E-09
Oasl2	1.757023	2.54E-08	3.22E-09
Adgrg2	1.744161	3.04E-05	5.65E-06
Sulf2	1.725825	1.86E-05	3.35E-06
Serpinb9	1.704544	6.96E-05	1.36E-05
Ccdc108	1.690896	1.2E-09	1.34E-10
Cyr61	1.681824	6.36E-05	1.24E-05
Il6ra	1.67516	2.57E-05	4.74E-06
Gm6961	1.669575	0.000423	9.44E-05
Dbh	1.66523	1.16E-13	9.14E-15
Slc7a11	1.648288	5.31E-81	6.11E-83
Glt8d2	1.640458	0.000035	6.58E-06
Klc3	1.61193	0.000156	3.24E-05
Cybrd1	1.605721	1.43E-05	2.54E-06
Itgb4	1.600904	3.62E-21	1.81E-22
Gfpt2	1.600393	0.000232	4.96E-05
Cish	1.600393	8.1E-08	1.09E-08
Col6a1	1.593424	1.95E-26	7.76E-28
Col14a1	1.584963	0.00083	0.000196
Nrip2	1.552372	4.39E-13	3.65E-14
Cdsn	1.548832	1.24E-56	2.2E-58
Apol9a	1.526368	0.000452	0.000101
Ttc12	1.520832	1.07E-14	7.84E-16
Itga2	1.520832	0.000452	0.000101
9630028I04Rik	1.51625	0.000167	3.47E-05
Gpr12	1.49322	1.23E-16	7.95E-18
Cdhr2	1.478047	0.000215	4.57E-05
Efna1	1.476814	3.2E-06	5.16E-07
Nthl1	1.469983	5.24E-06	8.72E-07
Pkhd1l1	1.467126	2.62E-14	1.98E-15
Ctla2a	1.462397	5.73E-81	6.62E-83
Tmem173	1.455109	5.45E-27	2.12E-28
Isg20	1.432959	1.79E-07	2.5E-08

Hist2h3c2	1.430719	1.99E-10	2.05E-11
N4bp2l1	1.423986	1.37E-12	1.18E-13
Slc41a3	1.409054	4.06E-10	4.36E-11
Fkbp9	1.408831	1.19E-61	1.89E-63
Grap2	1.389042	0.000512	0.000116
Fth1	1.380215	0	0
Lpin3	1.378512	9.77E-05	1.97E-05
Mest	1.377382	0	0
Lhx1	1.376895	1.54E-15	1.07E-16
Nfix	1.371256	3.36E-14	2.57E-15
Ctla2b	1.36434	3.95E-06	6.45E-07
Crhbp	1.359403	0.00049	0.00011
Lama4	1.353881	1.1E-238	3.2E-241
Rgmb	1.353637	4.28E-06	7.02E-07
Car6	1.336363	1.06E-06	1.61E-07
Hspa5	1.333103	0	0
Zfp57	1.329889	4.42E-72	6.05E-74
Arl4d	1.321268	5.53E-15	4E-16
Lrrc55	1.31946	9.81E-09	1.19E-09
Cbfa2t3	1.317926	2.53E-66	3.75E-68
Cdc42se2	1.31056	1.8E-101	1.6E-103
Ryk	1.302837	2.89E-97	2.7E-99
Abcc3	1.295456	1.25E-54	2.34E-56
Kdelr3	1.293006	6.84E-05	1.34E-05
Pla2g4b	1.289507	3.68E-07	5.33E-08
Trim21	1.2868	1.11E-18	6.37E-20
Prkg2	1.286304	5.12E-05	9.82E-06
Rbm18	1.28102	2.72E-58	4.69E-60
Wfs1	1.276687	4.35E-47	9.48E-49
Phtf1os	1.274175	0.000171	3.57E-05
Catsperd	1.270089	2.03E-05	3.69E-06
Serpina1d	1.255257	9.56E-25	4.05E-26
BB557941	1.25464	0.00025	5.37E-05
Zbtb20	1.24952	1.11E-09	1.23E-10
Avpi1	1.236892	4.19E-12	3.77E-13
Gm15085	1.234206	2.4E-06	3.81E-07
Hpse	1.231044	1.28E-06	1.97E-07
Plch2	1.23078	3.23E-06	5.22E-07
Ptgs1	1.211463	7.89E-45	1.78E-46
Fam167a	1.20581	8.35E-07	1.26E-07
Creld2	1.198048	7.83E-78	9.37E-80
Slc19a2	1.193195	2.66E-65	4.03E-67
Crk	1.189864	5.4E-112	4.4E-114
Ypel3	1.188457	5.46E-43	1.29E-44

Rnf8-cmtr1	1.180572	8.68E-06	1.49E-06
Angpt2	1.172837	0.000359	0.000079
S100a11	1.14943	0.000225	0.000048
Avil	1.148677	1.79E-08	2.23E-09
Socs2	1.145082	6.73E-30	2.31E-31
Ttll9	1.142958	0.000795	0.000187
Mamdc2	1.142353	2.45E-08	3.08E-09
Dapp1	1.141012	2.18E-06	3.44E-07
Adora1	1.136462	4.65E-10	5.02E-11
Nxf7	1.128577	2.14E-44	4.92E-46
Igf2r	1.123382	6.53E-05	1.27E-05
Spry1	1.121251	7.69E-45	1.73E-46
Cpox	1.115024	1.18E-37	3.14E-39
Ifitm3	1.114155	1.01E-05	1.76E-06
Irgm2	1.112741	5.57E-12	5.06E-13
Tle6	1.109451	4.55E-07	6.65E-08
Fgf1	1.106915	2.34E-20	1.22E-21
Chpf2	1.098326	4.19E-41	1.03E-42
Ier3	1.097618	1.12E-30	3.7E-32
Mafb	1.088809	1.15E-35	3.25E-37
F2rl2	1.083249	6.88E-78	8.2E-80
B4galnt2	1.082462	0.000881	0.000209
Icosl	1.079072	1.8E-06	2.81E-07
Manf	1.071215	9.6E-119	7.2E-121
Gstm7	1.069605	9.74E-06	1.68E-06
Btg2	1.058344	1.15E-27	4.32E-29
Nap1l5	1.057995	1.1E-44	2.5E-46
Tmprss6	1.057844	1.11E-05	1.94E-06
Sema3f	1.057371	2.17E-23	9.7E-25
Adamts10	1.056584	5.74E-08	7.54E-09
Rapgef3	1.055142	7.22E-05	1.42E-05
Calr	1.054194	0	0
Pbxip1	1.048363	1.12E-08	1.37E-09
Cyb561	1.047693	4.48E-61	7.23E-63
Pdia4	1.042939	1.7E-184	6.8E-187
Stard5	1.038459	8.39E-42	2.03E-43
Arntl	1.036005	1.31E-22	6.06E-24
Asic4	1.033088	1.96E-22	9.16E-24
Tnfrsf23	1.032421	0.000654	0.000152
Srp14	1.024541	1.8E-38	4.68E-40
Ssx2ip	1.019562	2.14E-26	8.51E-28
Ero1lb	1.015733	4.49E-13	3.74E-14
Gadd45a	1.00931	2.06E-20	1.07E-21
Phlda1	1.00827	3.36E-76	4.28E-78

Ephb4	1.008174	1.58E-05	2.82E-06
Fbll1	1.003958	0.000366	8.06E-05
Sertad2	1.003128	1.28E-47	2.78E-49

S3 Table. List of genes downregulated in TET3-silenced, compared to control N2A cells. Genes with a statistical significance of P1≤0.0001 are listed. FDR: false discovery rate.

**Symbol**	**log2FoldChange (N2aTET3/N2aControl)**	**FDR**	**Pvalue**
Rps27rt	-10.5507	6.11E-25	2.56E-26
0610010B08Rik	-7.79442	1.28E-49	2.68E-51
Gm10096	-7.65105	1.18E-08	1.45E-09
Gm5071	-7.62936	7.96E-10	8.78E-11
Gm28042	-7.40088	1.13E-42	2.72E-44
Gm6644	-7.2854	4.06E-10	4.36E-11
Gm14305	-7.14975	5.43E-11	5.31E-12
Gm14819	-7.10852	9.38E-06	1.62E-06
Gm10230	-7.10852	9.38E-06	1.62E-06
Gm14632	-7.10852	9.37E-06	1.62E-06
Gm8923	-6.80735	6.57E-07	9.77E-08
Gm3317	-6.74147	6.03E-09	7.2E-10
Pbld1	-2.45943	5.55E-08	7.27E-09
Tnr	-2.43812	9.56E-21	4.86E-22
Trf	-2.43296	9.72E-05	1.96E-05
Tshr	-2.39993	1.26E-09	1.41E-10
Gfra1	-2.38941	3.53E-75	4.56E-77
Porcn	-2.30875	0.000163	3.39E-05
Pdpr	-2.27436	4.67E-32	1.48E-33
Zeb2os	-2.26303	0.000231	4.94E-05
Rgs5	-2.24793	0.000136	0.000028
Gm20604	-2.04217	1.54E-33	4.61E-35
Slit2	-1.92979	4.7E-127	3.3E-129
Uhmk1	-1.91882	1.05E-37	2.79E-39
Lgr5	-1.90332	2.13E-08	2.66E-09
Gm12070	-1.8997	9.33E-08	1.26E-08
Bnc2	-1.89038	3.74E-16	2.5E-17
Mmp15	-1.88982	2.2E-132	1.5E-134
Tet3	-1.8573	3.3E-183	1.4E-185
Rel	-1.83354	3.8E-06	6.2E-07
Caly	-1.81835	9.36E-07	1.42E-07
Lcor	-1.78526	7.57E-12	6.98E-13
Pcdhga3	-1.78427	4.35E-05	8.27E-06
Fst	-1.774	8.06E-14	6.3E-15
Mir124a-1hg	-1.77138	1.85E-06	2.89E-07
Clcn5	-1.7576	2.96E-46	6.51E-48
Pcdh15	-1.75002	3.91E-13	3.22E-14

Higd2a	-1.74238	4.94E-60	8.24E-62
D830031N03Rik	-1.72792	4.18E-07	6.09E-08
Fbxo47	-1.72348	0.000449	0.0001
Nrp1	-1.71617	0	0
Disc1	-1.69213	5.2E-15	3.74E-16
Myh7b	-1.69032	1.32E-06	2.04E-07
Ttc28	-1.6471	1.1E-183	4.8E-186
Wnt5a	-1.62209	3.62E-32	1.14E-33
Gpr137c	-1.61471	6.13E-10	6.69E-11
Setd7	-1.61262	2.9E-181	1.3E-183
Greb1l	-1.52356	9.59E-08	1.3E-08
Bc1	-1.52143	4.1E-112	3.4E-114
Lcmt2	-1.51457	5.57E-05	1.07E-05
AI661453	-1.49354	5.99E-07	8.87E-08
Endog	-1.47685	1.06E-07	1.45E-08
Slc9a7	-1.46949	1.22E-05	2.13E-06
Zdhhc22	-1.46362	7.2E-06	1.22E-06
Shox2	-1.44276	1.23E-75	1.58E-77
Naip7	-1.44057	7.92E-05	1.56E-05
Cntn2	-1.43296	5.82E-09	6.93E-10
Id4	-1.42396	1.59E-40	3.95E-42
Wwp1	-1.42033	3.23E-38	8.43E-40
Fchsd2	-1.41954	2.45E-75	3.14E-77
Dnajc22	-1.4112	0.000142	2.95E-05
Ppp1r12b	-1.40411	5.62E-11	5.51E-12
Sparcl1	-1.40169	6.29E-17	4E-18
Cbl	-1.40032	5.19E-28	1.92E-29
Tmem65	-1.37924	5.2E-103	4.6E-105
Megf10	-1.37246	2.07E-80	2.4E-82
Capn6	-1.36427	1.22E-65	1.83E-67
Dusp19	-1.34885	9.8E-09	1.19E-09
Pdlim3	-1.33381	1.1E-123	8E-126
Arhgap28	-1.33258	0.00025	5.38E-05
Fam171b	-1.33137	1.04E-65	1.56E-67
2500002B13Rik	-1.32925	0.000597	0.000138
Sv2c	-1.31791	3.7E-211	1.3E-213
Lrig1	-1.31074	7.37E-28	2.74E-29
Axl	-1.30625	1.27E-08	1.57E-09
Fam126b	-1.30541	1.24E-25	5.07E-27
1110001J03Rik	-1.29438	1.83E-13	1.47E-14
Ptprr	-1.28301	1.33E-46	2.93E-48
9130024F11Rik	-1.2713	0.000173	3.62E-05
Prelid2	-1.26843	4.89E-08	6.38E-09
Mapk6	-1.26712	9.9E-118	7.5E-120

Trim56	-1.25919	6.86E-08	9.11E-09
Gm15093	-1.24841	2.88E-20	1.51E-21
Trim71	-1.24793	2.38E-15	1.67E-16
Gm3558	-1.24462	1.79E-08	2.23E-09
Tbc1d4	-1.24226	7.66E-43	1.82E-44
Pcdhac1	-1.23879	3.29E-06	5.32E-07
H2-Q6	-1.23729	2.62E-07	3.73E-08
Dok3	-1.22677	4.81E-05	9.21E-06
Rasl10b	-1.22507	1.6E-16	1.04E-17
Mfsd4a	-1.2205	1.2E-06	1.84E-07
Unc79	-1.21658	1.08E-74	1.4E-76
Smad9	-1.21087	5.21E-49	1.09E-50
Tbx20	-1.21011	5.46E-45	1.23E-46
Ksr2	-1.20645	2.43E-05	4.47E-06
Fam135b	-1.20436	9.27E-05	1.86E-05
5033428I22Rik	-1.20271	7.54E-09	9.1E-10
Kbtbd11	-1.19855	1.14E-32	3.54E-34
Klhl11	-1.19417	5.64E-14	4.37E-15
Nlrp6	-1.18763	1.62E-06	2.51E-07
Plcxd3	-1.1858	3.63E-25	1.51E-26
Slc29a3	-1.18525	2.18E-27	8.31E-29
Megf9	-1.18458	3.46E-21	1.72E-22
Obscn	-1.18442	7.75E-10	8.52E-11
Ago2	-1.18268	1.8E-127	1.2E-129
Nrp2	-1.17267	1.7E-295	3.4E-298
Adssl1	-1.17172	7.04E-15	5.12E-16
Lgi4	-1.15948	7.74E-10	8.52E-11
Synpo2	-1.15673	2.35E-08	2.95E-09
Zbed6	-1.15666	2.06E-15	1.44E-16
Atp1a3	-1.1557	0	0
Zdhhc2	-1.15295	2.5E-205	9.2E-208
9030617O03Rik	-1.1527	4.86E-12	4.39E-13
Scn2a1	-1.14439	3.16E-20	1.66E-21
LOC102636514	-1.14417	4.35E-22	2.07E-23
Car12	-1.14412	1.92E-70	2.71E-72
Isca2	-1.14302	1.29E-20	6.63E-22
Evi5l	-1.1394	1.43E-14	1.06E-15
Pik3c2b	-1.13006	1.59E-07	2.22E-08
Pgm2l1	-1.12928	1.17E-18	6.72E-20
Pip4k2a	-1.12646	2.29E-33	6.92E-35
Dock5	-1.11818	2.49E-07	3.53E-08
Pdk1	-1.11722	1.17E-21	5.69E-23
Fzd2	-1.09761	7.64E-12	7.06E-13
Ago3	-1.09686	5.81E-05	1.12E-05

Tnks	-1.08785	1.9E-106	1.6E-108
Cdkl5	-1.08066	3.88E-10	4.15E-11
Ankrd52	-1.07899	1.2E-156	6E-159
Ppp1r3f	-1.078	2.03E-05	3.68E-06
Usp49	-1.07702	2.44E-13	1.99E-14
Plekha2	-1.0756	6.35E-74	8.41E-76
Slc41a2	-1.0739	5.07E-24	2.21E-25
Nbeal1	-1.06871	1.02E-07	1.39E-08
Dlg2	-1.06843	2.39E-19	1.33E-20
Hipk2	-1.06711	1.5E-09	1.69E-10
Tmem256	-1.06551	6.14E-23	2.81E-24
Atp10d	-1.06496	4.24E-46	9.39E-48
St6galnac5	-1.05355	0.000409	9.09E-05
Nav3	-1.05247	1.81E-06	2.83E-07
Agtpbp1	-1.0497	6.67E-29	2.39E-30
Elovl4	-1.0431	4E-31	1.31E-32
Gab2	-1.03884	5.32E-55	9.9E-57
Nr1d2	-1.03776	3.48E-89	3.49E-91
Brwd3	-1.03118	3.52E-18	2.1E-19
Zfp3	-1.02915	0.000307	6.65E-05
Crem	-1.02647	3.94E-07	5.72E-08
Tmem8b	-1.02623	2.19E-07	3.09E-08
Inpp5j	-1.02606	2.46E-09	2.82E-10
9330182L06Rik	-1.0204	4.86E-38	1.28E-39
Arl5b	-1.02023	6.64E-17	4.23E-18
F7	-1.01937	0.000256	5.51E-05
Prex1	-1.01648	8.89E-55	1.66E-56
Dock4	-1.01427	1.2E-108	9.9E-111
Loxl2	-1.00043	0	0
Ptchd2	-1	1.43E-09	1.61E-10
Usp29	-1	3.65E-05	6.88E-06

S4 Table. Differentially hydroxymethylated regions considered of statistical significance between control and TET3-silenced N2A cells within mRNA-associated promoter regions. These genes were hypo-hydroxymethylated in TET3-silenced cells. TSS: Transcriptional start site

GeneName	DMR_To_TSS	p-value
Aqp6	-1989	6.54E-05
Krt7	-1952	1.28E-05
Grik5	1299	0.000645
Abcc8	-727	0.000453
Atp6v1c1	647	0.000477
Fgf10	-878	0.000357
Rnf39	-1000	0.000911
Arhgef7	-964	0.000458
Smarcd2	1212	0.000977
Pou3f1	1515	0.000102
Usp13	535	0.000816
Rras2	1841	0.000576
Slc51a	1489	0.000782
Dmrtc1c2	1436	0.000399
Dmrtc1c1	1436	0.000399
Crmp1	-587	0.000684
Atp7b	1194	0.0004
Rnf216	-580	0.00077
Acp5	276	0.000977
Dgkz	-327	5.63E-05
Sema4a	-1528	0.000214
Nup93	1320	0.00037
Epas1	-163	0.000165
Dstyk	268	0.00077
Gm11570	1593	0.000461
Bahcc1	1784	0.0005
Phldb3	413	1.29E-06
Hoxc6	1649	7.76E-05
Adamts7	-397	0.000178
Ttbk1	-355	1.08E-05
Hoxb8	336	8.25E-05
Rgmb	606	0.000553
Pnlip	-944	0.000241
Appl2	-896	1.16E-05
Ifitm10	-531	0.000175

Jmjd8	-302	0.000229
Ddx19b	421	0.000473
Ppfibp2	-760	0.000754
Dact2	541	0.000323
Zfp266	1879	0.001
Myo1e	-1359	0.0006
Abtb1	-75	0.0006
Srsf11	54	0.000881
Creld1	1562	8.25E-05
Slc36a3	-1294	0.000143
Myg1	-1738	0.0006
Asic1	-1537	0.000365
Cyp2c40	1164	4.45E-05
Cdh3	528	1.7E-06
Bcl11b	964	0.00048
Hoxa3	1217	0.001
Fam13c	701	0.000753
Smad6	1099	0.000237
Clic6	214	0.000834
Kif21a	898	0.000427
Tanc2	505	9.29E-06
Mgll	-341	0.000759
Lypd2	1383	0.000118
Ttc34	-409	0.000195
Strada	-67	0.000541
Acot6	1677	0.000877
Pcdhb8	1090	0.000225
Tgds	-725	0.00092
Asic3	-240	0.000759
Rnf186	1389	1.24E-05
Meis1	789	0.000807
Pmvk	190	4.2E-05
Ptx3	474	0.000204
Olfr370	218	0.000136
Ctrc	1959	0.000267
Setd1a	1502	7.94E-06
Stx1a	-1381	0.000157
Nt5c2	-1221	0.000759
1700012A03Rik	-1497	0.000204
Klk13	-716	4.08E-06
Ttc36	-1749	0.000499
St8sia1	532	0.000102
Pcna	-670	0.00024
Gfm2	464	0.000764

Cyr61	595	4.34E-06
Ebag9	-1550	0.000693
P2ry14	-906	0.000379
1810062G17Rik	484	3.02E-06
Scn8a	-300	0.000666
Nanos2	1247	0.000664
Zfp54	-396	0.000171
Tex40	-470	0.000176
Csrnp2	-481	0.000176
Cldn14	-1963	5.98E-05
Tmprss9	315	0.000764
Tgfa	1850	0.000189
2510049J12Rik	-754	0.000189
E130003G02Rik	921	0.000987
Wbscr17	452	7.06E-05
Rnf146	-308	0.000995
Capn1	-305	6.75E-05
Pard6b	1057	0.000379
Eid2b	1785	0.000379
Nuak1	-1239	0.000667
Ncl	1285	0.000667
Slc35e3	-1062	0.000147
Nlgn1	1739	0.000667
Slc12a9	-688	0.000649
Gpr111	-1771	0.000764
Zhx2	-416	1.18E-06
Tmem140	1325	0.000898
Chmp7	-1280	0.000667
Scmh1	170	0.000667
Hmox1	233	0.000703
Aak1	564	0.000703
Nkpd1	840	4.17E-05
Kcng2	1174	4.17E-05
Snx6	1552	0.000301
Rnf222	-52	0.0002
Mamstr	1394	9.11E-05
Lhfpl2	655	9.11E-05
Gtf2ird2	1473	0.000181
Zfp703	-1635	2.02E-05
1700021F05Rik	1394	0.000166
Jam2	-322	4.17E-05
Gbas	-804	4.17E-05
Omp	-873	0.000114
Snupn	1417	0.000667

Papola	1009	0.000915
Rangap1	-441	0.000915
Ost4	-1932	0.000915
Det1	-79	0.000915
Gli1	549	0.000459
Gatc	1321	0.000459
Wdr74	1542	0.00085
Cybrd1	1397	0.00036
Pou6f1	15	9.11E-05
Tmem132e	-789	9.11E-05
Sec14l5	-728	0.000631
P2rx5	-359	0.000377
Ccdc107	886	0.00042
Tsku	-82	0.000358
Akap9	555	0.000192
Ubb	-43	0.000192
Six1	-778	0.000192
Zfp692	-1968	0.000299
Polr3e	1537	8.47E-05
Tmub1	696	0.00012
Acot11	871	4.11E-06
Tro	-974	0.000915
Lmo4	1742	0.000915
Des	-133	0.000915
Cxxc1	1839	0.000915
Agphd1	261	0.000915
Nfatc1	1283	0.000915
Kctd8	1069	0.000915
Bend6	961	0.000915
Atp6v1c2	-1200	0.000915
Tnpo1	1534	0.000968
Smtnl2	473	0.00046
Wasf1	1777	0.000439
Olfr284	119	0.000439
Sall3	1598	0.000511
Slc16a10	1214	0.000192
Gpr150	1366	0.00014
Btbd17	1512	4.03E-05
Fam132b	-129	7.53E-05
Gnptab	1519	1.27E-05
Hoxb5	-21	7.78E-06
Zfp85-rs1	-646	0.000744
Zfp777	-1376	0.000308
Zfp54	604	0.000887

Ywhaz	-548	0.000744
Wwc1	-1541	0.000887
Unkl	69	0.000137
Uchl1	920	0.000484
Uba7	-1426	0.000642
Tnfaip8l3	711	0.000137
Tbc1d30	1199	0.000951
Srsf4	-1848	5.78E-05
Slc6a6	1284	0.000744
Slc39a13	1701	2.41E-05
Sctr	-499	0.000308
Rc3h2	-1287	6.5E-05
Rab17	-513	0.000951
Pycr1	-70	0.000607
Pds5b	1694	0.000951
Pcdha12	-729	0.000744
Parp11	-62	1.78E-06
Paox	856	0.000744
Olfr366	940	0.000308
Olfr272	-248	0.000178
Odf2	1228	0.00045
Nr3c1	-1375	0.000744
Nipsnap1	28	0.000642
Nhp2l1	738	1E-05
Ncoa3	-986	0.00051
Mrps30	-1618	0.00045
Mpnd	-1720	3.63E-05
Mettl1	499	0.000744
Mcts2	-417	0.000308
Mb21d2	-1302	0.000887
Map7	-1669	0.000744
Map3k7cl	-949	0.00045
Maml1	1806	0.000308
Magi2	785	0.000181
Ly6e	-1280	0.000744
Lrrc8b	336	0.000744
Lfng	-790	0.000744
Lamb1	-303	6.5E-05
Kdm6b	-1855	0.000137
Kcnj11	867	0.000887
Il2rb	1136	5.71E-05
Hoxb5	1359	0.000951
Fabp1	-1937	0.000744
Dtnb	1220	0.000284

Dkk4	1288	0.000293
Ctbp2	1537	0.000887
Cnot6l	-390	0.000951
Cnksr3	1217	0.000744
Cldn22	1369	0.000642
Chrna4	147	0.000484
Cdh22	1787	0.000396
BC107364	1496	0.00045
Atxn7l1	-235	0.00051
Aldh1a3	57	0.000744
Ablim1	-768	0.000744
Ablim1	649	0.000744
Abca8b	-1064	0.000951
Abca5	-34	1E-05
Aaed1	-580	0.000744
9130204L05Rik	1505	0.000279
4930455F23Rik	1913	0.000951

S5 Table. Differentially hydroxymethylated regions considered of statistical significance between control and TET3-silenced N2A cells within mRNA-associated promoter regions. These genes were hyper-hydroxymethylated in TET3-silenced cells. TSS: Transcriptional start site.

GeneName	DhMR_To_TSS	p-value
Pgr	238	0.000955
Pfkm	761	0.000168
Ncmap	-1523	0.000511
Nab2	613	0.000955
Mybphl	-1060	0.000955
Inpp5a	632	0.000461
Gm7173	915	0.000955
Cela3b	-990	1.6E-06
Atp1a4	424	5.82E-05
Olfr902	-791	0.000759
Ulk4	1412	0.000225
1700037H04Rik	1300	0.000237
Cdr1	268	0.000735
Sec16b	425	9.72E-05

S5 Fig. Uncropped and unadjusted images. (A) Original blot shown in Fig 2E, (B) Original blot shown in Fig 4A

A

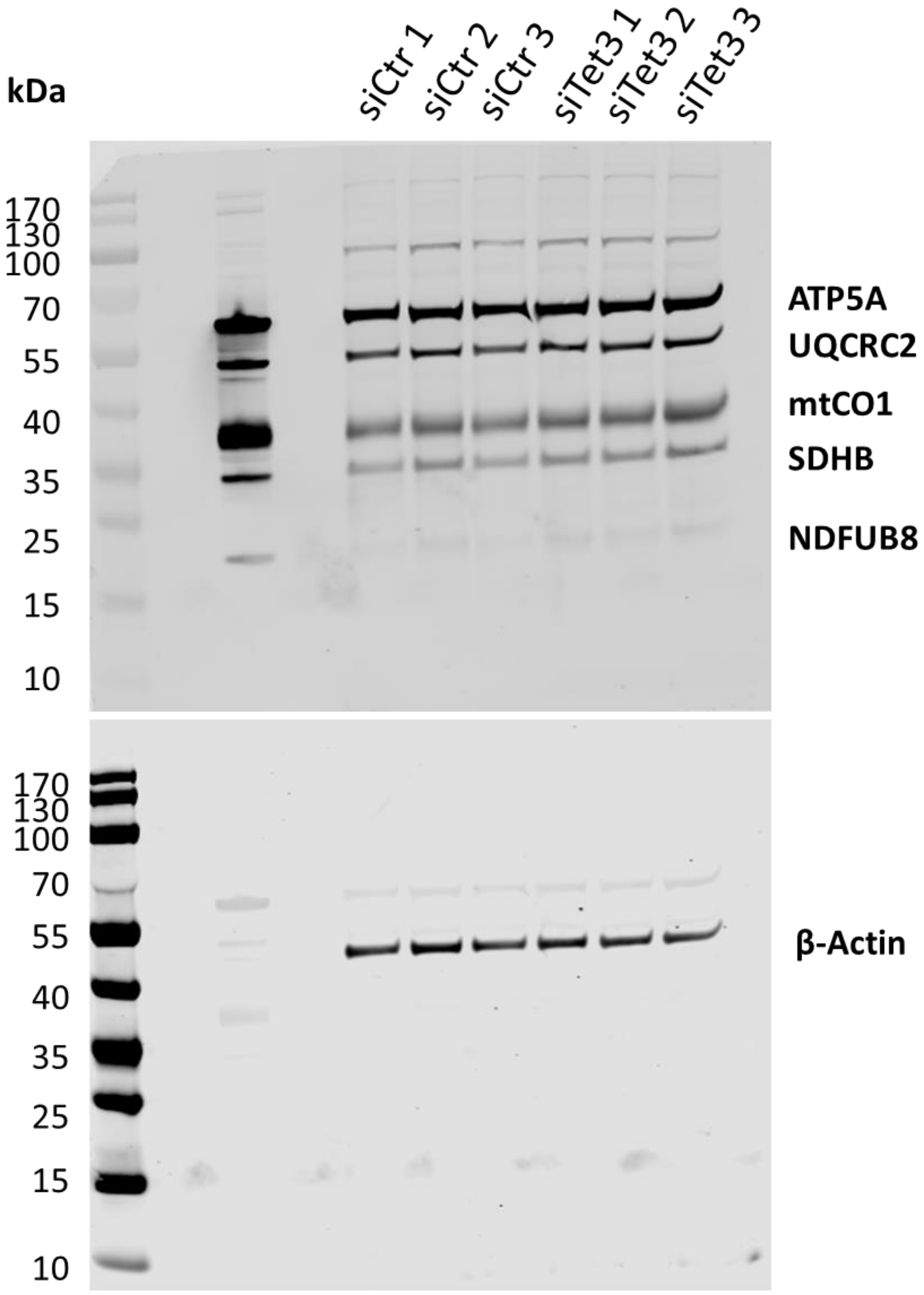

B

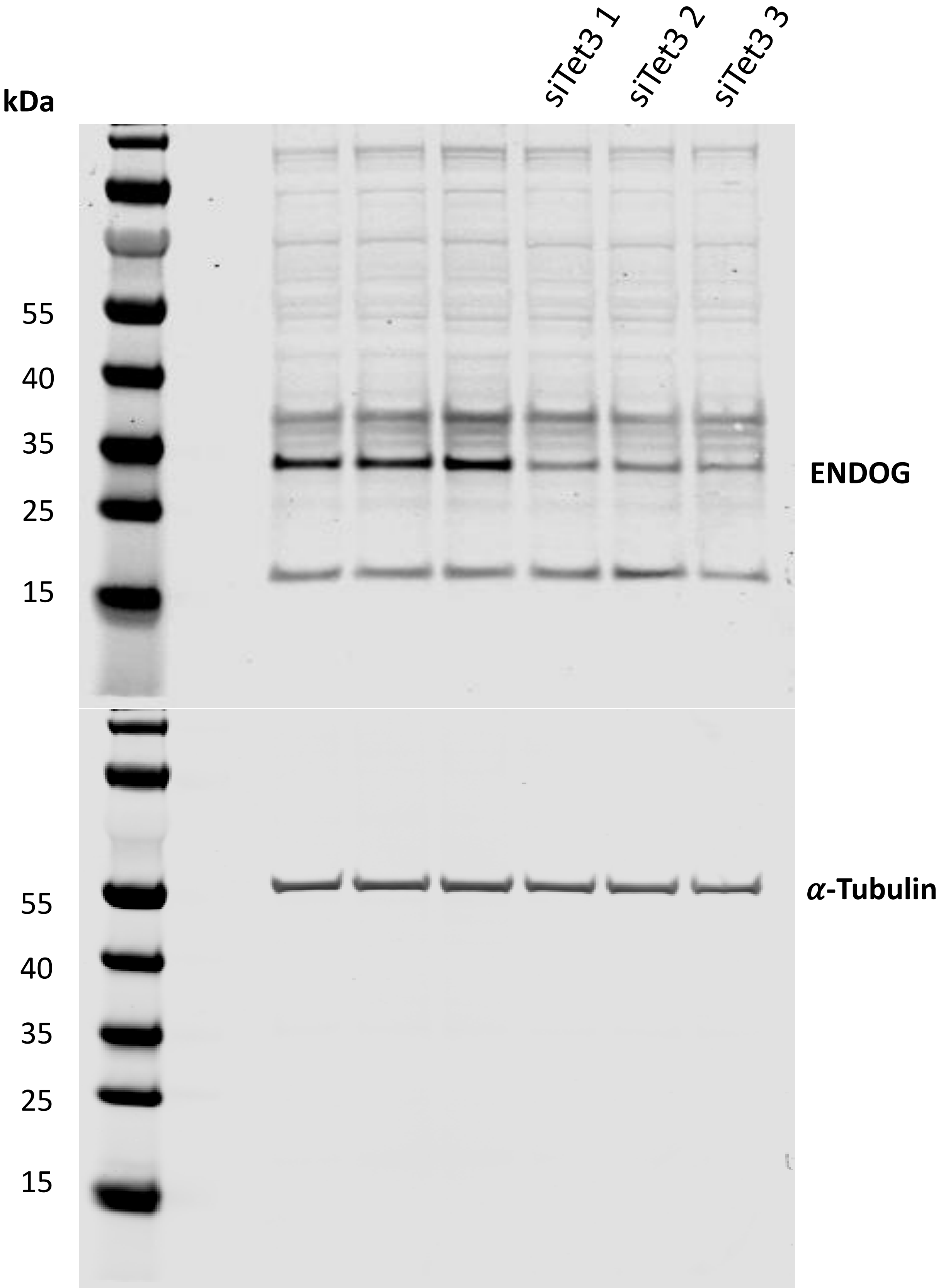
siTet3 1
siTet3 2
siTet3 3
kDa
55
40
35
25
15
ENDOG
55
40
35
25
15
α-Tubulin
