# Supplementary material for: TET3 is a positive regulator of mitochondrial respiration in Neuro2A cells
Source: PLoS One. 2024 Jan 16;19(1):e0294187. doi: 10.1371/journal.pone.0294187 (PMC10790995; doi:10.1371/journal.pone.0294187)
Supplement: Attachment — Submitted filename: Response to Reviewers.docx [file pone.0294187.s002.docx]

We would like to thank the reviewers for taking the time to review our manuscript and for their helpful suggestions.

We have now amended our manuscript as detailed below.

Reviewer #1: This study used pharmacological and genetic manipulations, as well as transcriptomic and biochemical analysis to investigate the role and targets of TET3 in N2A cell line. The result from RNA seq suggested that oxidative phosphorylation was identified as the most significantly down-regulated pathway by Tet3-silencing, consisted with the downregulation of oxygen consumption rate, the mRNA levels of nuclear- and mitochondrial encoded oxidative phosphorylation, and mitochondrial quality control genes. Among these genes, EndoG was identified as a direct target of TET3-catalytic activity at non-CpG methylated sites within its gene body. The authors indicated that the decrease in OxPhos may be due to dysfunctional mitochondria rather than direct transcriptional regulation of OxPhos genes by TET3. This work represents enriched data and a series of interesting observations. However, there are some major concerns regarding the interpretation of the results.

Major
1. The protein levels of each of the components assessed (ATP5A, UQCRC2, mtCO1, SDHB and NDFUB8) were found to be slightly increased upon Tet3-silencing, in contrast to the transcriptome data and the Q-PCR verification. Does Tet3-silencing enhance the protein synthesis or decrease the protein degradation? It is easy to assess by different inhibitors.

**Response: We fully concur that both enhanced (aberrant) protein synthesis and reduced protein degradation could result in the discrepancy between the changes in mRNA and protein levels of the OxPhos that we observed upon TET3 downregulation together with the decrease in mitochondrial bioenergetics. This point is now discussed (highlighted in the 4^th^ para of discussion). However, we have not, as yet, carried out any further experiments (using inhibitors) to investigate this further. We believe that due to the complex nature of the different pathways which regulate mitochondrial function this would prove quite challenging and time consuming. We believe that this kind of mechanistic study (based on our findings here) might be more informative (and potentially clinically relevant) if performed in primary neurons, which is beyond the scope of this paper.**

2. Figure 4 Restoring the full-length Tet3 or the kinase-dead mutants should be included to elucidate the specific role of TET3 on the EndoG regulation in N2a cells.

**Response: We identify EndoG as a catalytic target of TET3 as we show it to be upregulated by 5-azaC (suggesting it is regulated by methylation), to be downregulated by DMOG (indicating it to be regulated by the catalytic activity of a 2-OGDD enzyme), and to exhibit changes in 5-hmC upon TET3 downregulation. The rescue experiments proposed, using wild-type and kinase (catalytic?)-dead mutants would be inappropriate in our system, since we deplete TET3 expression using siRNAs, which would also degrade the “rescuing” gene expression. In addition, TET3 in mouse has both long and short isoforms, and it is not clear which would be the relevant isoform to test functionally in the rescue experiment. However, this is, of course a very interesting question with respect to the biochemistry of TET3, and determining which isoform is responsible for the catalytic activity on the EndoG promoter in a neuronal-like cell is something that we are currently aiming to determine.**

3. What is the functional relationship of TET3 and EndoG in regulating the mitochondrial respiration or oxidative phosphorylation in N2A cell? Synergistically or not? Does restore EndoG rescue the impaired phenotype by TET3 silencing?

**Response**: **The** **silencing of TET3 resulted in the significant downregulation of a number of genes, known to be involved in mitochondrial function (Fig 1E &F). One purpose of our study was to determine which genes might be regulated by the catalytic activity of TET3, and only EndoG fell into that category. However, the other genes regulated by other (non-catalytic/more downstream) mechanisms would also contribute to the functional dysregulation of the mitochondria. Thus, we do not suggest that EndoG misexpression alone is responsible for the loss of mitochondrial bioenergetics, or that Endo G expression alone would rescue the phenotype. Rather, we suggest that it may be involved. However, we now realise that this was not made sufficiently clear and we apologise. We have now included a paragraph at the end of the discussion to clarify this point.**

4. The title is misleading because N2A is neuroblastoma cell line.

**Response: We have now changed the title of the manuscript to make it clear that the study was performed in Neuro2A cells.**

5 “The decrease in OxPhos may be due to dysfunctional mitochondria rather than direct transcriptional regulation of OxPhos genes by TET3” is overstated.

**Response: We have changed this sentence (at the end of the abstract) to *“****Accordingly, we propose that aberrant mitochondrial homeostasis may contribute to the decrease in OxPhos, observed upon Tet3-downregulation in Neuro2A cells”.* **We believe that this makes it clearer that the changes in OxPhos observed may in part also result from the changes in OxPhos gene expression.**

Reviewer #2: This is a well written manuscript with some minor issues:

1. Please define "low glucose" in the methods, with mM concentration for the N2a cells.

**Response: This information is now included in the methods section.**

2. Please define the concentrations of drugs used in the seahorse assay (oligomycin, FCCP, and Rotenone/Antimycin A)

**Response: This information is now included in the methods section.**

3. In the results section page 6, 2nd paragraph there is a highlighted XXX which needs to be filled in with the correct information for the GEO accession number.

**Response: If our manuscript is accepted, we will provide the repository information. We have also informed the editor of this.**
